# Dying Words of Celebrated Persons

**Published:** 1880-03

**Authors:** 


### DYING WORDS OF CELEBRATED PERSONS.

*From "Bronchitis and Kindred Diseases."*

"Head of the army."—*Napoleon.*

"L'Isle D'Elbe, Napoleon."—*Josephine.*

"I must sleep now."—*Byron.*

"It matters little how the head lieth."—*Sir W. Raleigh.*

"Kiss me, Hardy."—*Lord Nelson.*

"Don't give up the ship."—*Lawrence.*

"I'm shot if I don't believe I'm dying."—*Chancellor Thurlow.*

"Is this your fidelity?"—*Nero.*

"Clasp my hand, my dear friend, I die."—*Alfieri.*

"Give Dayroles a chair."—*Lord Chesterfield.*

"God preserve the Emperor."—*Hayden.*

"The artery ceases to beat."—*Haller.*

"Let the light enter."—*Goethe.*

"All my possessions for a moment of time."—*Queen Elizabeth.*

"What! is there no bribing death."—*Cardinal Beaufort.*

"I have loved God, my father, and liberty."—*Mad. De Stael.*

"Be serious."—*Grotius.*

"Into thy hands, O Lord."—*Tasso.*

"It is small, very small indeed, (clasping her neck).—*Anne Boleyn.*

"I pray you, see me safe up, and for my coming down, let me shift for myself," (ascending the scaffold).—*Sir Thos. More.*

"Don't let that awkward squad fire over my grave."—*Robert Burns.*

"I feel as if I were to be myself again."—*Sir Walter Scott.*

"I resign my soul to God, and my daughter to my country. —*Jefferson.*

"Am I so far gone?"—*Niebuhr.*

"There is not a drop of blood on my hands."—*Frederick V., of Denmark.*

"Let me hear once more those notes which have so long been my solacement and delight."—*Mozart.*

"A dying man can do nothing easy."—*Franklin.*

"Let not poor Nelly starve."—*Charles II.*

"Let me die to the sounds of delicious music."—*Mirabeau.*

"The Lord reigns, let the earth rejoice."—*Rev. Dr. E. Corne-*

"Remorse."—*John Randolph of Roanoke.*

"Doctor, I think I am getter weaker, feel my pulse."—*John Newland Maffit.*

"Adieu, my beloved Cuba; adieu my brethren," (the instant before his execution).—*General Lopez.*

"Sister, I am weary, let us go home."—*Neander.*

"But even the log on the Delaware, has its care-taker."—*Dr. Joseph Parish.*

"How violent is this disorder, how very extraordinary it is!" —*Stephen Girard.*

"I forgive the authors of my death, and I pray that my blood may not fall upon France," (the moment before he was guillotined).—*Louis XVI.*

"I'm most gone."—*Rev. Andrew Todd.*

"It is well."—*Washington.*

"Independence for ever."—*Adams.*

"It is the last of earth."—*J. Q. Adams.*

"I wish you to understand the true principles of the government. I wish them carried out. I ask nothing more."—*Harrison.*

"I have endeavored to do my duty."—*Gen. Taylor.*

"Doctor, I am dying very hard, it seems as though I shall never get through with it."—*Vice President King.*

"I could wish this tragic scene were over."—*Quinn, the Actor.*

"God bless you all. A general good night."—*Dr. Chalmers.*

"I still live."—*Daniel Webster.*

"My son, don't leave me. I'm going soon."—*Henry Clay.*

